# Motility Performance of Thawed Spermatozoa of Bulls from the Tropics Throughout the Year

**DOI:** 10.3390/ani15162451

**Published:** 2025-08-21

**Authors:** Annie Y. Poclín-Rojas, Martin Daniel Arbaiza Barnechea, Gleni T. Segura Portocarrero, Gustavo Ampuero-Trigoso, Diana Bernilla Carrillo, Benjamín A. Depaz-Hizo, Ronald W. Vásquez-Tarrillo, Clavel Diaz-Quevedo, Hurley A. Quispe-Ccasa

**Affiliations:** 1 Laboratorio de Biotecnología Animal, Instituto Nacional de Innovación Agraria, Juan Guerra 22400, Peru; anniepoclinrojitas@gmail.com (A.Y.P.-R.); diani272829@gmail.com (D.B.C.); rvasqueztarrillo0609@gmail.com (R.W.V.-T.); 2 Estación Experimental Agraria El Porvenir, Instituto Nacional de Innovación Agraria, Juan Guerra 22400, Peru; bdepaz@inia.gob.pe (B.A.D.-H.); velcitadiaz@gmail.com (C.D.-Q.); 3 Dirección de Desarrollo Tecnológico Agrario, Instituto Nacional de Innovación Agraria, Lima 15024, Peru; arbaiza.b@gmail.com (M.D.A.B.); gustavoampuerotrigoso@gmail.com (G.A.-T.); 4 Facultad de Ingeniería Zootecnista, Agronegocios y Biotecnología, Universidad Nacional Toribio Rodríguez de Mendoza de Amazonas, Chachapoyas 01001, Peru

**Keywords:** *Bos indicus*, tropical livestock, cryopreservation, seasonal period, temperature-humidity index, sperm velocity

## Abstract

Under tropical conditions, seasonal variations may also influence the sperm characteristics of *Bos indicus*. The objective was to evaluate the motility of thawed sperm of bulls from the Peruvian tropics throughout the year. Over 24 months, 129 ejaculates were evaluated based on semen quality and subjected to slow horizontal freezing in 0.5 mL straws. After thawing, the individual, seasonal period, and season effect on motility and kinetic parameters were analyzed using a Sperm Class Analyzer^®^ (Microptic S.L.U., Barcelona, Spain). There was an individual effect on volume, motility, fresh concentration, and kinetic parameters when thawed. In the dry period, higher straight-line velocity (VSL) (*p* < 0.05) and beat cross frequency (BCF) were found than in the rainy period (*p* < 0.01). In summer and autumn, there was greater total motility, fast, circular routes, curvilinear velocity, average path velocity, VSL, amplitude of lateral displacement of the head, and BCF (*p* < 0.01). Greater volume and motility were found in winter and spring, but in summer and autumn, greater speed and vigor of movement were obtained in thawed sperm. The variation in annual climate patterns influences the seminal quality of bulls, and its effect needs to be assessed to propose adaptation strategies to climate change in tropical areas.

## Simple Summary

In the Peruvian tropical region, seasonal periods of rainfall and drought are generally distinguished, which do not necessarily coincide with the traditional spring, summer, autumn, and winter seasons. Recently, a shift in seasonal climate patterns has been suggested, which could influence the reproductive performance of zebu bulls. In this study, greater sperm motility vigor was observed in semen collected during the dry season. Meanwhile, during the summer and autumn, lower semen volume was recorded, but with higher motility vigor. This increased vigor was also observed in thawed sperm. The summer and autumn months (from January to June) were less hot than winter and spring (from July to December), likely as a consequence of global warming. It is necessary to analyze the effect of these environmental variations in order to develop adaptation strategies for breeding centers in tropical regions.

## 1. Introduction

Accelerated changes in weather patterns have been reported in tropical regions in recent years, creating more challenging conditions for livestock [1,2]. In tropical areas, scrotal temperature of bulls below 4–6 °C with respect to body temperature contributes to maintaining reproductive performance and seminal quality; however, climatic variations in the usual patterns of the year generate increases in temperature in atypical periods, which are capable of reducing the fertility of the bull [3,4]. *Bos indicus* bulls, such as the Gyr and Brahman breeds, are adapted to tropical climates and are more tolerant of high temperatures in the hottest periods of the year than *Bos taurus* breeds [5,6]; however, the increase in temperature during the hottest months can generate thermal stress, causing damage to the spermatogenic cells and defects in seminal quality [7]. It has been reported that higher ambient temperatures combined with elevated levels of relative humidity increase the temperature–humidity index (THI), negatively affecting testicular function, as well as sperm concentration, morphology, and motility [6,8]. THI values above 75 have been associated with reduced semen quality in bulls in tropical regions [1,2].

Motility is a very important characteristic of sperm function that is related to its fertilizing capacity and can be a good indicator of sperm viability and variations throughout the year [9]. Seasonal increases in THI may also compromise semen cryotolerance and, consequently, its motility and viability [5]; moreover, freezing has negative impacts on sperm structure and function too, as evidenced by reduced motility and movement speed [10]. Therefore, the objective of the study was to evaluate the motility performance of thawed sperm from Gyr and Brahman bulls throughout the year, under tropical conditions in northeastern Peru.

## 2. Materials and Methods

### 2.1. Study Location

The study was carried out from February 2022 to January 2024 at the Artificial Insemination (AI) center of the El Porvenir Agrarian Experimental Station, San Martín, Peru. The area is located at 223 m above sea level, between 6°35′20.5″ south latitude and 76°19′5.7″ west longitude. The environmental conditions correspond to a humid tropical climate, with a temperature of 25 °C, 72.9% relative humidity, and 1200 mm of precipitation (El Porvenir Meteorological Station, Servicion Nacional de Meteorología e Hidrología SENAMHI).

### 2.2. Experimental Design

Under a completely randomized design, 129 ejaculates were analyzed over 24 months from the 4 bulls available at the AI center (32 ejaculates per bull as mean). Ejaculates with good sperm concentration and motility were frozen with a uniform cryopreservation protocol. The variation factors were individual, seasonal period (rainy period: 21 September to 20 March and dry period: 21 March to 20 September), and season effect (spring: 21 September to 20 December, summer: 21 December to 20 March, autumn: 21 March to 20 June, and winter: 21 June to 20 September). The interaction effect was analyzed between the individual and the season effect (4 × 4).

### 2.3. Animals and Care

All experiments have been conducted as per the Animal Research: Reporting of In Vivo Experiments (ARRIVE 2.0) guidelines and Law 30407 on animal protection and welfare from the Peruvian Government. Four bulls: 03 Gyr > 93.8% purebred (Camilo-Gyr or CA-GY, Nativo-Gyr or NA-GY, and Paraiso-Gyr or PA-GY) and 1 Brahman 100% purebred (Solis-Brahman or SO-BR), aged 4 to 9 years, with a healthy reproductive clinical history, dewormed, good body condition, sexually active, and subjected to weekly semen collection by electroejaculation, were housed in individual stalls with uniform management, health, and feeding. The daily feed consisted of a green forage mix of *Pennisetum purpureum* × *Pennisetum typhoides*, *Pennisetum purpureum* × *Pennisetum glaucum*, and *Pennisetum* sp., provided twice daily. In addition, 4 kg of a concentrated feed based on corn, rice flour, and mineral salt were provided daily, and fresh water was freely available. Every 15 days, 8 mL of ADE vitamins (Vigantol^®^ ADE, Bayer, Leverkusen, Germany) and 15 mL of minerals (selenium, phosphorus, and zinc) (Fertimin Se^®^, Agrovet, Tlaquepaque, Mexico) were administered intramuscularly, and 60 g of mineral salts (Fosvimin^®^, Montana, Lima, Peru) were added to food three times a week.

### 2.4. Semen Collection and Cryopreservation

Semen collections were performed once a week in the morning using the electroejaculation method, as the bulls were not early trained for semen collection via artificial mounting and had a relatively nervous temperament. An automatic electroejaculator (Autojac V3^®^, Neovet, Uberaba, MG, Brazil) equipped with a rectal transducer was used. The ejaculates were collected in graduated tubes and subjected to a quality assessment. Sperm concentration was analyzed in a photometer (SD1M, Minitube, Tiefenbach, Germany), and mass motility was estimated with a score from 0 to 5, and individual motility under phase contrast microscopy. Good-quality ejaculates were pre-diluted (1:1) in OptiXcell^®^ medium (IMV Technologies, L’Aigle, France) in a 34 °C water bath flask. After 10 min, the remaining diluent was added to achieve 30 million motile sperm per 0.5 mL straw. Sperm motility was checked, and the sperm were packaged in 0.5 mL straws.

The packaged straws were placed inside tempered water and refrigerated at 5 °C for 16 h. The straws were subjected to slow horizontal freezing for an initial drop from 0 to −140 °C for 7 min (20 °C/min) on liquid nitrogen vapors. Once −140 °C was reached, the straws were completely immersed in liquid nitrogen and stored in a cryogenic tank until thawing. The straws were thawed in water at 37 °C for 30 s, and the motility variables were evaluated.

### 2.5. Motility

Motility was analyzed in a Sperm Class Analyzer^®^ or SCA^®^ (Microptic, Barcelona, Spain) equipped with a phase contrast microscope (CX31, OLYMPUS, Tokyo, Japan), camera (BASLER, acA780-75gc, Ahrensburg, Germany), heated stage, and 10X objective. The particle detection size was set at 5 to 70 µm^2^, and 50 frames per second were captured. The following variables were determined in three random fields, as average [11]: curvilinear velocity (VCL), straight-line velocity (VSL), average path velocity (VAP), progressive motility (MP), non-progressive motility (MNP), total motility (MT), and classification according to speed: fast (>50 µm/s), medium (>25 µm/s), and slow (>10 µm/s). Moreover, the following kinetic parameters were determined: linearity (LIN), straightness (STR), wobble (WOB), amplitude of lateral displacement of the head (ALH), beat cross frequency (BCF), spermatozoa with a circular route (RC), hyperactivated sperm (VCL > 150 µm/s, ALH > 3.5 µm), and spermatozoa with mucus penetration capacity (ALH > 1.25 µm, STR > 80%, VAP > 25 µm/s).

### 2.6. Statistical Analysis

The goodness of fit of all variables was analyzed with the Kolmogorov–Smirnov test and the Levene test. According to the individual, breed, seasonal period, and season effect, data with normal distribution were analyzed using ANOVA and Duncan’s test (*p* < 0.05), while data without normal distribution were analyzed with the Mann–Whitney U test and Kruskal–Wallis test (*p* < 0.05). The interactive effect between individual × season was analyzed using the Bonferroni adjustment (*p* < 0.05) in SPSS v.15.0.

## 3. Results

The volume of ejaculate, mass motility, and sperm concentration showed significant differences between individuals. SO-BR had the highest ejaculate volume, mass motility, and sperm concentration (*p* < 0.01) (Table 1). After thawing, NA-GY and PA-GY stood out in MP, MT, and fast (*p* < 0.01). NA-GY showed the lowest MNP (*p* < 0.01) and highest RC (*p* < 0.05), and there were no differences between CA-GY, PA-GY, and SO-BR. SO-BR showed a higher percentage of slow spermatozoa (*p* < 0.05). Significant differences also were found in VCL (*p* < 0.05), VAP (*p* < 0.05), and VSL (*p* < 0.01) between individuals, but there were no differences in STR, LIN, and WOB (*p* > 0.05) (Table 2). ALH and BCF also varied significantly, where PA-GY stood out over SO-BR (*p* < 0.05). In addition, the number of hyperactivated spermatozoa and mucus penetration capacity stood out in NA-GY (*p* < 0.01).

**Figure 1:**
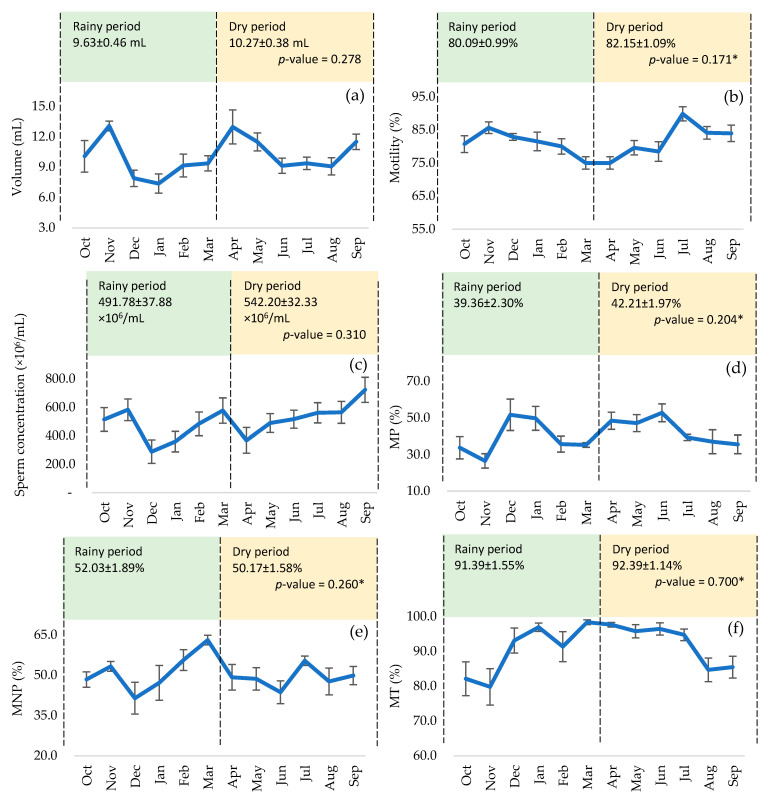
Semen quality and motility of thawed bovine spermatozoa according to the seasonal period: (**a**) volume, (**b**) motility, (**c**) concentration, (**d**) progressive motility, (**e**) non-progressive motility, and (**f**) total motility. (*) Analysis with the non-parametric test.

**Figure 2:**
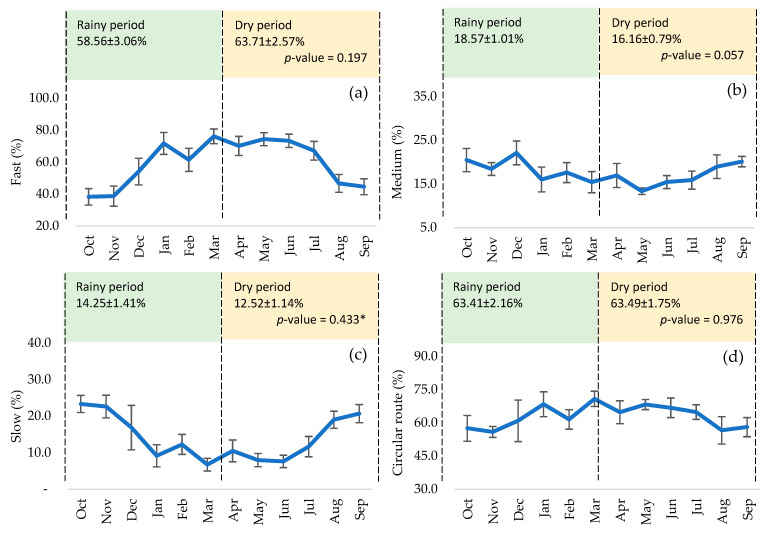
Motility of thawed bovine spermatozoa according to the seasonal period: (**a**) fast, (**b**) medium, (**c**) slow, and (**d**) circular route. (*) Analysis with the non-parametric test.

**Figure 3:**
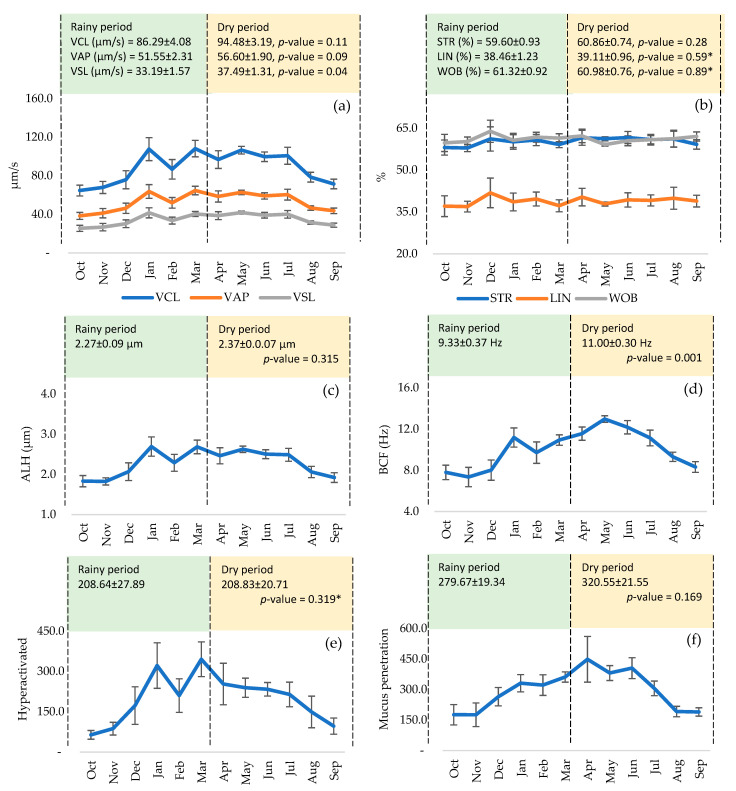
Kinetic parameters of thawed bovine spermatozoa according to the seasonal period: (**a**) curvilinear velocity, average path velocity, and straight-line velocity; (**b**) straightness, linearity, and wobble; (**c**) amplitude of lateral displacement of the head; (**d**) beat cross frequency; (**e**) hyperactivated; and (**f**) mucus penetration. (*) Analysis with a non-parametric test.

No significant differences were found in volume between the seasonal period (*p* > 0.05), although peaks were reached from October to November and April to May (Figure 1a). Individual motility peaked from July to September (Figure 1b) and sperm concentration from September to November and February to March (Figure 1c), although there were no significant differences between both periods (*p* > 0.05). Similarly, after thawing, there was no significant effect on MP (Figure 1d), MNP (Figure 1e), and MT (Figure 1f), although they showed higher peaks from December to March and April to June. Fast sperm (Figure 2a) and RC (Figure 2d) maintained relatively stable curves from January to July, and medium (Figure 2b) and slow sperm (Figure 2c) showed peaks from July to September and October to December, with no significant differences between periods (*p* > 0.05). There were no significant differences in VCL, VAP (Figure 3a), and ALH (Figure 3c), although the curves remained stable from January to July. However, a significant effect was found on VSL (Figure 3a) and BCF (Figure 3d), highlighting the dry period over the rainy period (*p* < 0.05 and *p* < 0.01), with a peak in BCF from April to June. STR, LIN, and WOB were stable throughout the year, with no significant influence of the seasonal period (*p* > 0.05) (Figure 3b). The number of hyperactivated spermatozoa (Figure 3e) and mucus penetration capacity (Figure 3f) also did not vary significantly, but showed peaks from January to March and April to June, respectively.

The higher maximum temperatures and THI were found in winter and spring, while more rainfall and humidity were recorded in summer and autumn. According to the season, higher volume of ejaculate in autumn and spring (*p* < 0.05) and higher individual motility in winter and spring (*p* < 0.01) were found (Table 3). Mass motility did not vary significantly between groups (*p* < 0.05), and sperm concentration was higher in winter and spring, although not significantly. After thawing, significant variation was found in MT, fast, medium, slow (*p* < 0.01), and RC (*p* < 0.05). In summer and autumn, higher rates of MT and fast sperm were found, while in spring and winter, there were higher rates of medium and slow sperm. In addition, the highest RC was found in autumn compared to winter and spring (*p* < 0.05). The highest MP was also found in the autumn, although not significant (Table 3). In autumn and summer, there was higher VCL and ALH (*p* < 0.01), and in autumn, there was higher VAP, VSL, and BCF (*p* < 0.01) than in winter and spring (Table 3). The number of hyperactivated spermatozoa was higher in summer and autumn, and the mucus penetration capacity was higher in autumn (*p* < 0.01).

The individual × season interactive effect was analyzed, and no interaction was found for most variables except for sperm concentration (*p* < 0.01) (Table S1). Sperm concentration in CA-GY increased in summer and autumn, whereas in PA-GY it decreased during these seasons. In summer and winter, the sperm concentrations of both bulls tend to approach each other but diverge in subsequent seasons (Figure S1). Although the concentration levels varied, the sperm concentration curves for SO-BR and LA-GY were similar and increased throughout the seasons.

## 4. Discussion

In tropical conditions, the effect of genotype on semen quality is known in *Bos taurus* and *Bos indicus*. In this study, Brahman bulls had higher spermiogram values than Gyr bulls in whole ejaculates, mainly volume, mass motility, and concentration, although they had similar individual motility rates before dilution. After thawing, the kinetic parameters were higher in Gyr bulls; however, a larger number of animals would reinforce a difference between breeds. In other reports, climatic factors influence the sexual behavior of Brahman bulls [12] and lower motility in the semen of Braford bulls at the end of summer, probably due to the end of the breeding season [13]. Individual genetics, age, nutrition, thermoregulation, and testicular biometry are key factors that determine the seminal characteristics of bulls [14,15]; therefore, variations in fresh, diluted, and thawed semen were to be expected.

According to the seasonal period, an average of 34.37 ± 0.14 °C, 72.49 ± 0.36%, and 1232 mm was found in the rainy period, and 33.10 ± 0.13 °C, 73.99 ± 0.34%, and 921.3 mm in the dry period, of maximum temperature, humidity, and rainfall, respectively. High temperatures influence spermatogenesis and epididymal maturation and may reduce sperm motility and morphology in susceptible breeds [16]. *Bos indicus* shows better semen parameters than *Bos taurus* due to its better thermoregulatory capacity [8]. There are reports about the effect of the seasonal period in tropical regions on the semen quality of *Bos indicus* [17,18,19]. Seasonal periods are associated with variations in precipitation, temperature, humidity, and forage availability. In the humid tropical region of northeastern Peru, the scarce differentiation between seasons suggests the use of seasonal periods for agricultural purposes. The rainy season generally runs from October to March, and the dry season from April to September; however, neither period showed significant differences in seminal volume, concentration, or sperm motility in this study. Landaeta-Hernández et al. [7] in Venezuela classified the seasonal periods into warm-dry (January to late April), transition (late April to early June), warm-humid (late June to September), and cool-rainy (October to December); the warm-humid period showed higher sperm motility and concentration. Another report found no effect of the seasonal period on scrotal circumference or seminal quality, although the rainy season reduced libido and mating ability, and the transition period from summer to the onset of the rain increased them [12]. The rainy and dry seasonal periods of this study recorded similar levels of maximum temperature and humidity, which could explain their reduced effect on semen quality. The rains from October to December were delayed, and high temperatures from June to September were prolonged, resulting in similar average values in both periods. However, adequate nutritional conditions and comfort in the AI center could have contributed to reducing the environmental effect.

Analysis by season showed a marked effect on semen quality. In southern Brazil, Koivisto et al. [6] analyzed the semen quality of *Bos indicus* according to season. They found greater motility in winter, and greater progressive motility in summer and autumn. In this study, autumn and spring showed the highest ejaculate volume, and winter and spring showed the highest motility and concentration, despite higher maximum temperatures and lower rainfall. In males, exposure temperatures above 27 to 32 °C can cause testicular degeneration and, consequently, defects in spermatogenesis and semen quality [20]. In Braford bulls, higher fresh sperm motility (80.45 to 86.81%) was reported when the THI ranged from 82.81 to 85.67, compared to a lower motility (74.54%) when the index increased to 87.91 [13]. THI values below 88 correspond to a risk of severe stress, but values above 88.1 represent severe stress with a risk of death [21]. Gyr bulls showed a negative association between environmental indices related to thermal stress and volume, concentration, motility, and sperm viability [8,22]. *Bos indicus* experiences less damage to the testicular parenchyma from thermal stress compared to *Bos taurus* because it is better adapted and regulates temperature in the testes more effectively [23]. In addition, thermal stress in the hottest months negatively affects the Sertoli cells and the Leydig cells, plasma testosterone concentration, and spermatogenesis, which decreases sperm concentration and motility and increases morphological defects [24,25,26]. The increase in cortisol levels in a thermal stress event could also affect testosterone concentrations; both hormones compete for the precursors cholesterol and pregnenolone for their synthesis. However, low correlations were reported between these two indicators [27,28], so the individual capacity for thermoregulation would be crucial in this aspect.

After thawing, Gyr individuals showed greater MP, MT, fast, and CR, and Brahman showed greater MNP and slow. Some reports presented similar motility in thawed semen from Gyr and *Bos taurus*, with no significant differences between individuals [29,30]. Furthermore, VCL, VSL, VAP, ALH, and BCF values in Gyr sperm showed greater movement vigor than Brahman. Airani et al. [30] found similar VCL but higher VSL and VAP (60.64 and 67.53 µm/s, respectively), and Pathak et al. [31] found lower motility, VCL, VSL, VAP, LIN, STR, and BCF in Gyr. The higher volume, concentration, and mass motility in Brahman did not result in higher motility after freezing, suggesting greater freezability of Gyr semen. The higher number of hyperactivated sperm and mucus penetration capacity may suggest greater fertility in thawed Gyr semen, because of the greater progressiveness and speed. The greater progressiveness and speed of thawed sperm are correlated with binding to the zona pellucida, penetration rate, and pronucleus formation [32]. However, the study of breed cryotolerance could be addressed in a future study with a larger number of animals per breed.

The dry period showed higher VSL and BCF, probably due to lower temperatures from April to June. The high temperatures of the rainy period could contribute to generating thermal stress and lower-quality spermatozoa evident after freezing. VSL and BCF show the vigor and linear progressiveness of the spermatozoa to reach the fertilization site. According to season, summer and autumn showed higher MT, fast, RC, VCL, VAP, VSL, ALH, BCF, hyperactivated sperm, and mucus penetration capacity than other seasons; in addition, these seasons recorded the lowest temperatures and THI values. In contrast, winter and spring showed high percentages of sperm with medium and slow velocities and high temperatures and THI values. There are some studies on the effect of the season on the quality and freezability of semen in tropical cattle [33,34]. The season influences the freezability of semen since batches made in the summer are of lower quality. During the summer, high temperatures and humidity can generate thermal stress even in adapted cattle, triggering lower semen quality [6,18]. Thermal stress generates an increased production of reactive oxygen species (ROS) in semen. Sperm bovine membranes are susceptible to lipid peroxidation by free radicals, because they are rich in polyunsaturated fatty acids, which causes damage to the biomolecules, structure, and motility [35,36]. Above 34.5 °C, metabolic rate and oxygen demand increase, leading to hypoxia, tissue oxidative stress, and ROS production [37,38]. Thermal stress also affects mitochondrial energy production through the loss of mitochondrial membrane potential and electron leaks in oxidative phosphorylation [39,40]. However, in *Bos indicus*, a lower production of ROS and adequate activity of glutathione peroxidase/reductase were reported, which is useful for reducing the effects of thermal stress in semen [22,35]. Unlike the literature, in this study, we found greater negative effects in winter and spring, probably due to the delay in spring rainfall, and they were more frequent in autumn, and temperatures remained high in winter and dropped in summer. In recent years, there have been accelerated changes in climate patterns due to global warming, generating variations in the usual climate of the annual seasons, such as atypical increases in temperature in winter and spring since 2012 (Figure S2) and delays in precipitation [2,4].

The interactive effect of individual × season was significant for sperm concentration, suggesting an individual adaptation according to season. Landaeta-Hernández et al. [7] found an interaction between genotype and seasonal period on volume, concentration, and mass motility. They highlighted the sperm concentration of *Bos indicus* compared to *Bos taurus* in the warm-dry period. The interaction between genotype and environment influences the fertility of bulls and manifests itself as seasonality in their semen quality, even in breeds more tolerant to tropical climates [5,6,35]. The variation in semen quality according to seasons may be due to genetic differences in the adaptation of breeds to tropical climates [41]. Although Landaeta-Hernandez et al. [7] reported that the interaction also influences the success of semen freezing, in this study, we found no effects on motility after thawing, probably due to the number of bulls.

Monitoring the environmental impact on semen quality is crucial for proposing adaptation strategies for production systems in response to climate change, especially in tropical regions. Future research involving a larger population of bulls trained and collected using the artificial vagina method, as well as simultaneous monitoring in other tropical regions, microscopic sperm evaluations, fertility, and the association with genes related to heat stress protein expression and cryotolerance, could help clarify these findings.

## 5. Conclusions

In summary, a significant effect was found in most of the seminal quality in fresh and thawed semen. The seasonal period only influenced VSL and BCF of thawed spermatozoa, highlighting the dry period (21 March to 20 September), probably due to the alteration of the usual climatic patterns between rainy and dry periods. However, the season markedly influenced the quality of fresh and thawed semen, with unexpectedly better freezability aptitudes in summer (21 December to 20 March) and autumn (21 March to 20 June), highlighting total motility, VCL, VAP, VSL, ALH, BCF, hyperactivated, and mucus penetration, probably due to the more comfortable climatic conditions compared to winter and spring. These findings show that semen quality coincides with the most comfortable climatic conditions in the tropics, but these vary according to environmental patterns and commonly established time limits. The evaluation of the effect of the variation of environmental patterns on bulls is useful to understand the adaptation of cattle and to propose strategies for the adaptation of productive systems in the tropics.

## Figures and Tables

**Table 1:** Semen quality and motility of thawed spermatozoa of Gyr and Brahman bulls according to individual and breed.

ID	n	Volume (mL)	MM	MI (%)	Concent. (×10^6^/mL)	MP (%)	MNP (%)	MT (%)	Fast (%)	Medium (%)	Slow (%)	Circular Route (%)
CA-GY	43	8.9 ± 0.4 ^b^	2.9 ± 0.1 ^bc^	83.4 ± 1.2	468.0 ± 40.9 ^b^	39.5 ± 2.5 ^bc^	52.9 ± 2.1 ^a^	92.4 ± 1.6 ^ab^	62.4 ± 3.4 ^a^	16.5 ± 1.1	13.5 ± 1.7 ^ab^	63.0 ± 2.5 ^b^
NA-GY	21	9.2 ± 0.7 ^b^	2.8 ± 0.1 ^c^	78.3 ± 1.9	278.4 ± 41.4 ^c^	54.9 ± 3.6 ^a^	41.9 ± 3.5 ^b^	96.8 ± 0.7 ^a^	67.6 ± 3.1 ^a^	19.1 ± 1.4	10.0 ± 1.4 ^b^	71.6 ± 2.6 ^a^
PA-GY	28	9.7 ± 0.6 ^b^	3.1 ± 0.1 ^ab^	79.5 ± 1.4	472.7 ± 48.2 ^b^	44.8 ± 3.4 ^b^	49.1 ± 2.9 ^a^	93.8 ± 2.0 ^a^	68.8 ± 4.3 ^a^	14.8 ±1.3	10.2 ± 1.9 ^b^	64.6 ± 3.0 ^ab^
SO-BR	37	12.0 ± 0.6 ^a^	3.2 ± 0.1 ^a^	81.6 ± 1.6	751.7 ± 29.2 ^a^	31.8 ± 1.8 ^c^	55.5 ± 1.3 ^a^	87.3 ± 2.0 ^b^	51.1 ± 3.9 ^b^	18.9 ± 1.3	17.3 ± 1.6 ^a^	58.5 ± 2.4 ^b^
*p*-value		<0.001	0.001 **	0.084	<0.001	<0.001	0.002	0.001 **	0.005	0.072	0.013	0.018
Total	129	10.0 ± 0.3	3.0 ± 0.1	81.2 ± 0.8	519.5 ± 24.6	40.9 ± 1.5	51.0 ± 1.2	91.9 ± 0.9	61.4 ± 2.0	17.2 ± 0.6	13.3 ± 0.9	63.5 ± 1.4

MM: mass motility; MI: individual motility; Concent.: sperm concentration; MP: progressive motility; MNP: non-progressive motility; MT: total motility. Different superscript letters ^(a, b, c)^ in columns indicate significant differences at *p* < 0.05 and *p* < 0.01 levels. (**) Analysis with a non-parametric test.

**Table 2:** Kinetic parameters of thawed bovine spermatozoa according to individual and breed.

ID	VCL (µm/s)	VAP (µm/s)	VSL (µm/s)	STR (%)	LIN (%)	WOB (%)	ALH (µm)	BCF (Hz)	Hyperactivated	Mucus Penetration
CA-GY	95.7 ± 5.0 ^a^	58.0 ± 2.9 ^a^	38.5 ± 2.0 ^a^	60.7 ± 1.1	39.5 ± 1.4	61.3 ± 1.0	2.4 ± 0.1 ^ab^	10.1 ± 0.4 ^ab^	228.7 ± 33.2	298.4 ± 21.5 ^bc^
NA-GY	87.4 ± 4.3 ^ab^	52.1 ± 3.1 ^ab^	33.1 ± 2.4 ^ab^	57.5 ± 1.5	36.1 ± 1.7	59.5 ± 1.3	2.3 ± 0.1 ^ab^	10.4 ± 0.5 ^ab^	204.5 ± 37.5	394.1 ± 45.0 ^a^
PA-GY	100.6 ± 5.7 ^a^	59.3 ± 3.2 ^a^	38.8 ± 2.1 ^a^	61.4 ± 1.4	39.1 ± 1.6	60.5 ± 1.1	2.6 ± 0.1 ^a^	11.6 ± 0.5 ^a^	256.3 ± 40.2	345.8 ± 34.5 ^ab^
SO-BR	79.6 ± 4.1 ^b^	47.6 ± 2.3 ^b^	31.1 ± 1.5 ^b^	60.5 ± 0.9	39.4 ± 1.5	62.4 ± 1.2	2.1 ± 0.1 ^b^	9.3 ± 0.5 ^b^	152.0 ± 22.9	221.4 ± 20.1 ^c^
*p*-value	0.015	0.013	0.008	0.181	0.485	0.429	0.017	0.016	0.183 **	0.001
Total	90.8 ± 2.6	54.3 ± 1.5	35.6 ± 1.0	60.3 ± 0.6	38.8 ± 0.8	61.1 ± 0.6	2.3 ± 0.1	10.3 ± 0.3	208.7 ± 16.9	302.2 ± 14.8

VCL: curvilinear velocity; VAP: average path velocity; VSL: straight-line velocity; STR: straightness; LIN: linearity; WOB: wobble; ALH: amplitude of lateral displacement of the head; BCF: beat cross frequency. Different superscript letters ^(a, b, c)^ in columns indicate significant differences at *p* < 0.05 and *p* < 0.01 levels. (**) Analysis with a non-parametric test.

**Table 3:** Semen quality, motility, and kinetic parameters of thawed bovine spermatozoa according to season.

Variables	Winter (21 June–20 September)	Spring (21 September–20 December)	Summer (21 December–20 March)	Autumn (21 March–20 June)	*p*-Value
n	37	25	33	34	
Volume (mL)	9.60 ± 0.45 ^ab^	10.83 ± 0.67 ^a^	8.72 ± 0.58 ^b^	11.01 ± 0.61 ^a^	0.017
MM	3.09 ± 0.08	2.96 ± 0.10	2.97 ± 0.09	3.10 ± 0.09	0.754 **
MI (%)	85.62 ± 1.50 ^a^	82.80 ± 1.00 ^a^	78.03 ± 1.47 ^b^	78.38 ± 1.33 ^b^	<0.001
Concent. (×10^6^/mL)	596.70 ± 42.66	508.72 ± 58.98	478.94 ± 50.03	482.88 ± 47.59	0.249
MP (%)	37.69 ± 2.75	38.84 ± 4.05	39.76 ± 2.70	47.14 ± 2.63	0.093
MNP (%)	50.65 ± 2.06	47.21 ± 2.38	55.68 ± 2.65	49.65 ± 2.46	0.110
MT (%)	88.34 ± 1.78 ^b^	86.04 ± 2.62 ^b^	95.44 ± 1.55 ^a^	96.79 ± 0.95 ^a^	<0.01
Fast (%)	53.32 ± 3.59 ^b^	45.52 ± 3.82 ^b^	68.45 ± 3.73 ^a^	75.02 ± 2.57 ^a^	<0.001
Medium (%)	18.09 ± 1.16 ^a^	20.57 ± 1.21 ^a^	17.06 ± 1.48 ^ab^	14.05 ± 0.94 ^b^	0.005
Slow (%)	16.91 ± 1.61 ^a^	19.96 ± 2.16 ^a^	9.93 ± 1.48 ^b^	7.73 ± 1.16 ^b^	<0.001 **
Circular route (%)	59.40 ± 2.67 ^b^	58.96 ± 3.36 ^b^	66.78 ± 2.72 ^ab^	67.94 ± 1.99 ^a^	0.027
VCL (µm/s)	84.16 ± 4.30 ^b^	71.05 ± 3.86 ^b^	97.84 ± 5.82 ^a^	105.71 ± 3.96 ^a^	<0.001
VAP (µm/s)	50.77 ± 2.59 ^b^	42.78 ± 2.41 ^c^	58.20 ± 3.19 ^ab^	62.93 ± 2.40 ^a^	<0.001
VSL (µm/s)	33.98 ± 1.83 ^b^	27.77 ± 1.84 ^c^	37.29 ± 2.14 ^ab^	41.32 ± 1.68 ^a^	<0.001
STR (%)	60.75 ± 1.13	58.95 ± 1.59	60.09 ± 1.11	60.99 ± 0.95	0.662
LIN (%)	39.65 ± 1.55	38.56 ± 1.99	38.38 ± 1.58	38.52 ± 1.10	0.922
WOB (%)	61.61 ± 1.23	61.36 ± 1.55	61.28 ± 1.14	60.29 ± 0.87	0.978 **
ALH (µm)	2.15 ± 0.09 ^b^	1.95 ± 0.09 ^b^	2.50 ± 0.12 ^a^	2.62 ± 0.08 ^a^	<0.001
BCF (Hz)	9.76 ± 0.41 ^b^	7.87 ± 0.44 ^c^	10.44 ± 0.48 ^b^	12.35 ± 0.31 ^a^	<0.001
Hyperactivated	152.62 ± 25.24 ^b^	119.08 ± 26.44 ^b^	276.48 ± 41.27 ^a^	270.00 ± 30.41 ^a^	<0.001 **
Mucus penetration	238.11 ± 19.41 ^c^	207.84 ± 26.74 ^c^	334.09 ± 23.44 ^b^	410.26 ± 33.81 ^a^	<0.001 **
Max. temperature (°C)	34.37 ± 0.16 ^c^	35.13 ± 0.19 ^d^	33.51 ± 0.18 ^b^	31.75 ± 0.15 ^a^	<0.001 **
Humidity (%)	71.16 ± 0.40 ^a^	71.58 ± 0.47 ^a^	73.52 ± 0.54 ^b^	77.01 ± 0.47 ^c^	<0.001 **
THI	88.08 ± 0.19 ^c^	89.30 ± 0.24 ^d^	87.23 ± 0.20 ^b^	85.13 ± 0.17 ^a^	<0.001 **
Accumulated rainfall (mm)	296.5	511.9	720.1	624.8	

MM: mass motility; MI: individual motility; Concent.: sperm concentration; MP: progressive motility; MNP: non-progressive motility; MT: total motility; VCL: curvilinear velocity; VAP: average path velocity; VSL: straight-line velocity; STR: straightness; LIN: linearity; WOB: wobble; ALH: amplitude of lateral displacement of the head; BCF: beat cross frequency; THI: temperature–humidity index. Different superscript letters ^(a, b, c, d)^ in rows indicate significant differences at *p* < 0.05 and *p* < 0.01 levels. (**) Analysis with a non-parametric test.

## Data Availability

The raw data supporting the conclusions of this article will be made available by the authors on request.

## Supplementary Materials

The following supporting information can be downloaded at https://www.mdpi.com/article/10.3390/ani15162451/s1. Figure S1: Interactive effect of individual × season on sperm concentration of bulls in the Peruvian tropics. SPSS v.15.0; Figure S2: Evolution of maximum temperature in the last 20 years (El Porvenir Meteorological Station, SENAMHI); Table S1: Interactive effect of individual × season on semen quality, motility, and kinetic parameters of thawed bovine spermatozoa.
